# Neural correlates of childhood trauma with executive function in young healthy adults

**DOI:** 10.18632/oncotarget.20051

**Published:** 2017-08-07

**Authors:** Shaojia Lu, Fen Pan, Weijia Gao, Zhaoguo Wei, Dandan Wang, Shaohua Hu, Manli Huang, Yi Xu, Lingjiang Li

**Affiliations:** ^1^ Department of Psychiatry, The First Affiliated Hospital, Zhejiang University School of Medicine, Key Laboratory of Mental Disorder’s Management of Zhejiang Province, Hangzhou, Zhejiang, China; ^2^ Department of Child Psychology, The Children's Hospital, Zhejiang University School of Medicine, Hangzhou, Zhejiang, China; ^3^ Mental Health Institute of The Second Xiangya Hospital, Key Laboratory of Psychiatry and Mental Health of Hunan Province, Central South University, Changsha, Hunan, China; ^4^ Department of Psychiatry, Shenzhen Kangning Hospital, Shenzhen, Guangdong, China

**Keywords:** childhood trauma, default mode network, fractional amplitude of low frequency fluctuation, functional connectivity, executive function

## Abstract

The aim of this study was to investigate the relationship among childhood trauma, executive impairments, and altered resting-state brain function in young healthy adults. Twenty four subjects with childhood trauma and 24 age- and gender-matched subjects without childhood trauma were recruited. Executive function was assessed by a series of validated test procedures. Localized brain activity was evaluated by fractional amplitude of low frequency fluctuation (fALFF) method and compared between two groups. Areas with altered fALFF were further selected as seeds in subsequent functional connectivity analysis. Correlations of fALFF and connectivity values with severity of childhood trauma and executive dysfunction were analyzed as well. Subjects with childhood trauma exhibited impaired executive function as assessed by Wisconsin Card Sorting Test and Stroop Color Word Test. Traumatic individuals also showed increased fALFF in the right precuneus and decreased fALFF in the right superior temporal gyrus. Significant correlations of specific childhood trauma severity with executive dysfunction and fALFF value in the right precuneus were found in the whole sample. In addition, individuals with childhood trauma also exhibited diminished precuneus-based connectivity in default mode network with left ventromedial prefrontal cortex, left orbitofrontal cortex, and right cerebellum. Decreased default mode network connectivity was also associated with childhood trauma severity and executive dysfunction. The present findings suggest that childhood trauma is associated with executive deficits and aberrant default mode network functions even in healthy adults. Moreover, this study demonstrates that executive dysfunction is related to disrupted default mode network connectivity.

## INTRODUCTION

Childhood trauma, including a spectrum of sexual, physical and emotional forms of abuse, as well as physical or emotional neglect, is highly prevalent and associated with risk for poor health outcomes in childhood and throughout the life course [1]. In recent years, increasing studies have focused on the long-term consequences of childhood trauma and a growing body of evidence suggests a link between childhood trauma and higher risk for cognitive impairments [2], especially executive dysfunction [3], in later life. It has been demonstrated that exposure to childhood trauma is often associated with poorer performance of executive function in patients with psychiatric disorders [4], individuals with ultra-high risk for psychosis [5], and even healthy school-aged children [6]. Moreover, a previous study has also revealed that childhood trauma may be specifically correlated with the subsequent development of cognitive symptoms of major depression [7]. Nevertheless, to date, the neural underpinnings of this consistently replicated relationship remain poorly understood.

Recently, resting-state functional magnetic resonance imaging (rs-fMRI) studies of samples characterized by a history of childhood trauma enable us to evaluate the neurobiological correlates of adverse early life experiences. In particular, functional neuroimaging studies have suggested that childhood trauma are associated with altered functional connectivity (FC) within- and between intrinsic neural networks [8]. A prominent finding is reduction of FC in the default mode network (DMN) [9, 10], while other key findings include disruptions in emotional processing networks [11], executive network [12], salience network [13], and amygdala-DMN, as well as insula-hippocampus connectivity [14]. In accordance with these findings, our prior study also reported that childhood trauma, in the absence of psychiatric diagnosis, was associated with altered DMN, cerebellum-DMN, and insula-DMN connectivity, along with regional homogeneity (ReHo) changes in the inferior parietal lobule (IPL), superior temporal gyrus (STG), insula, cerebellum, and middle temporal gyrus [15]. Meanwhile, Philip et al. (2013) used ReHo approach based on whole brain analysis and found a correlation of brain regional dysfunction in the IPL and STG with early life stress as well [16], suggesting that childhood trauma not only impacts FC but also brain regional activity.

Disturbances in intrinsic brain function have already been suggested to contribute to cognitive impairments observed in patients with psychiatric disorders [17]. It has been demonstrated that altered DMN connectivity is associated with executive processing deficits in patients with bipolar disorder [18] and Alzheimer’s disease (AD) [19]. Simultaneously, executive deficits in patients with major depressive disorder (MDD) have been repeatedly identified to be impacted by a dysfunction of prefrontal cortical regions [20]. However, such associations in subjects with childhood trauma are not clearly elucidated until now. Interestingly, limited task-related fMRI studies have revealed a number of functionally aberrant cortical and subcortical regions in subjects with childhood trauma during paradigms examining executive functions, such as working memory task [21] and response-inhibition task [22]. This could be interpreted as that brain functional abnormalities might also be implicated in the pathophysiology of childhood trauma related executive impairments.

Based on the above considerations, we present here a study trying to detect the long-term impacts of childhood trauma on executive performance and resting-state brain function in a group of young healthy adults and especially to investigate the associations between these two aspects in such a sample. In this study, the fractional amplitude of low frequency fluctuation (fALFF) method [23] was introduced to assess brain regional spontaneous activity. Then, selecting the identified group differences of fALFF as seed regions, the seed-based FC analysis [24] was used to evaluate interregional temporal connectivity. The fALFF approach is an advanced technique to measure local fluctuations in neuronal activity, rather than generalized neuronal activity, which can provide more specific index of low frequency oscillatory phenomena [25]. While coupled fALFF and FC analyses may allow us to completely evaluate brain intra-regional activity and interregional connectivity.

## RESULTS

### Demographics and clinical measures

As shown in Table 1, the two groups of subjects did not differ with respect to age, gender, educational level, self-rating anxiety scale (SAS) score, and self-rating depression scale (SDS) score. As we would expect, the two experimental groups differed on levels of Childhood Trauma Questionnaire (CTQ) and its sub-scales except sexual abuse. In maltreated subjects, the most common aspect of childhood trauma experience was emotional neglect (17, 70.8%); a proportion of 62.5% (15) of traumatic subjects experienced at least two forms of childhood trauma exposures.

**Table 1:** Demographic characteristics of all subjects (*n* = 48)

	CTE group *n =* 24 means (SD)	Non-CTE group *n =* 24 means (SD)	t/χ^2^	*p*-values
Age (Years)	21.5 (3.98)	21.5 (3.69)	−0.075	0.940
Gender (Male/Female)	9/15	9/15	0.000	1.000
Educational level (Years)	14.0 (1.30)	14.7 (1.92)	−1.407	0.166
SDS score	36.2 (6.06)	34.5 (5.30)	1.014	0.316
SAS score	34.0 (4.51)	32.0 (4.78)	1.430	0.160
Mean FD (mm)	0.11 (0.04)	0.10 (0.03)	0.835	0.408
CTQ score				
Emotional abuse	9.21 (2.36)	6.21 (1.22)	5.539	0.000
Physical abuse	7.83 (2.93)	5.71 (1.33)	3.324	0.002
Sexual abuse	5.46 (0.83)	5.38 (0.58)	0.403	0.689
Emotional neglect	15.2 (3.28)	7.38 (2.65)	9.094	0.000
Physical neglect	10.2 (2.72)	5.63 (0.93)	7.821	0.000
Total	47.9 (6.08)	30.2 (4.63)	11.38	0.000
CTE, n (%)				
Emotional abuse	2 (8.33)			
Physical abuse	8 (33.3)			
Sexual abuse	0 (0)			
Emotional neglect	17 (70.8)			
Physical neglect	14 (58.3)			
Multiply Exposures	15 (62.5)			
Single Exposure	9 (37.5)			

CTE, Childhood Trauma Exposures; CTQ, Childhood Trauma Questionnaire; FD, Framewise Displacement; SAS, Self-rating Anxiety Scale; SD, Standard Deviation; SDS, Self-rating Depression Scale.

#### Neuropsychological tests

As compared with the control group, individuals with childhood trauma had more preservative errors and less finished categories in Wisconsin Card Sorting Test (WCST). In Stroop Color Word Test (SCWT), the two groups exhibited significant difference in SCWT-A task. However, performance on Trail-making test (TMT) did not differ between two groups. For more details, please refer to Table 2. Correlation analyses revealed that scores of SCWT-A and completed categories in WCST were negatively (*r* = −0.289 ∼ −0.424, *p* = 0.003 ∼ 0.046), while preservative errors in WCST were positively (*r* = 0.395 ∼ 0.420, *p* = 0.003 ∼ 0.005) associated with CTQ scores (CTQ total, emotional neglect, and physical neglect scores) in the whole sample.

**Table 2:** Results of neuropsychological assessment of all subjects (*n* = 48)

	CTE group *n =* 24 means (SD)	Non-CTE group *n =* 24 means (SD)	*t*	*p*-values
WSCT				
TT	47.5 (7.79)	46.9 (1.93)	1.007	0.319
CT	27.5 (9.24)	32.2 (8.46)	−1.841	0.072
TE	20.0 (9.97)	14.8 (9.36)	1.881	0.066
PE	13.0 (6.63)	8.71 (6.05)	2.320	0.025
RE	7.04 (5.03)	6.04 (4.31)	0.739	0.463
Categories	3.50 (1.82)	4.67 (1.71)	−2.290	0.027
SCWT A	103.4 (16.5)	113.2 (13.7)	−2.239	0.030
SCWT B	74.8 (14.8)	79.3 (12.9)	−1.122	0.268
SCWT C	44.0 (10.3)	47.8 (9.63)	−1.320	0.193
SCWT interference	30.7 (9.75)	31.4 (9.26)	−0.258	0.798
TMT A (s)	29.9 (7.89)	31.7 (7.70)	−0.781	0.439
TMT B (s)	63.3 (16.0)	59.9 (13.5)	0.792	0.433

CTE, Childhood Trauma Exposures; CT, Correct Trials; PE, Preservative Errors; RE, Random Errors; SD, Standard Deviation; SWCT, Stroop Color Word Test; TE, Total Errors; TMT, Trail-making Test; TT, Total Trials; WSCT, Wisconsin Card Sorting Test.

#### fALFF analysis

**Figure 1:**
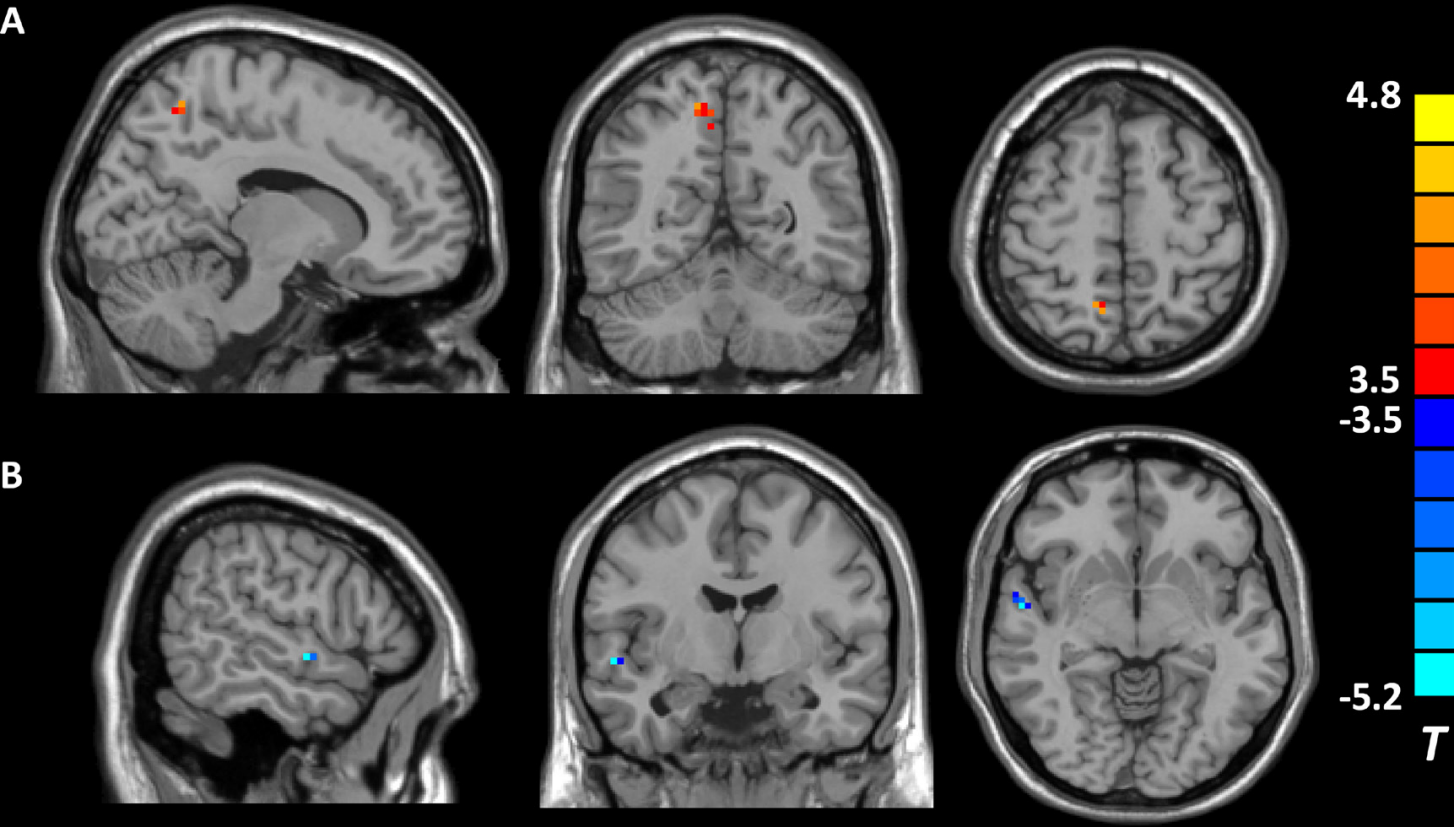
Brain regions showing different fALFF values between individuals with and without childhood trauma (*p* < 0.05, AlphaSim corrected) Hot and cold colors indicate increased and decreased fALFF in individuals with childhood trauma, respectively, compared with subjects without childhood trauma. (**A**) the right precuneus; (**B**) the right superior temporal gyrus.

As compared with subjects without childhood trauma, individuals with adverse experiences in childhood shared altered activity in two DMN regions, increased fALFF in the right precuneus, as well as decreased fALFF in the right STG (see Table 3 and Figure 1). There were positive correlations found between fALFF in the right precuneus and CTQ scores, including CTQ total (*x* = 6, *y* = −57, *z* = 57, *cluster* = 10, *T* = 4.00, *r* = 0.595), emotional neglect (*x* = 6, *y* = −57, *z* = 48, *cluster* = 7, *T* = 4.42, *r* = 0.600), and emotional abuse scores (*x* = 6, *y* = -57, *z* = 57, *cluster* = 9, *T* = 4.27, *r* = 0.571) in the whole sample. However, no association was found between cognitive tests and fALFF changes in the present study.

**Table 3:** Brain regions showing altered fALFF values in CTE individuals as compared with subjects without CTE

Brain region	Hemisphere	Cluster size	*T* value	MNI coordinate		
				*x*	*Y*	*z*
Increased						
Precuneus	R	14	4.30	12	−57	57
Decreased						
STG	R	10	−5.22	57	−6	−6

(*p* < 0.05, AlphaSim corrected). CTE, Childhood Trauma Exposures; MNI, Montreal Neurological Institute; STG, Superior Temporal Gyrus.

#### FC analysis

**Figure 2:**
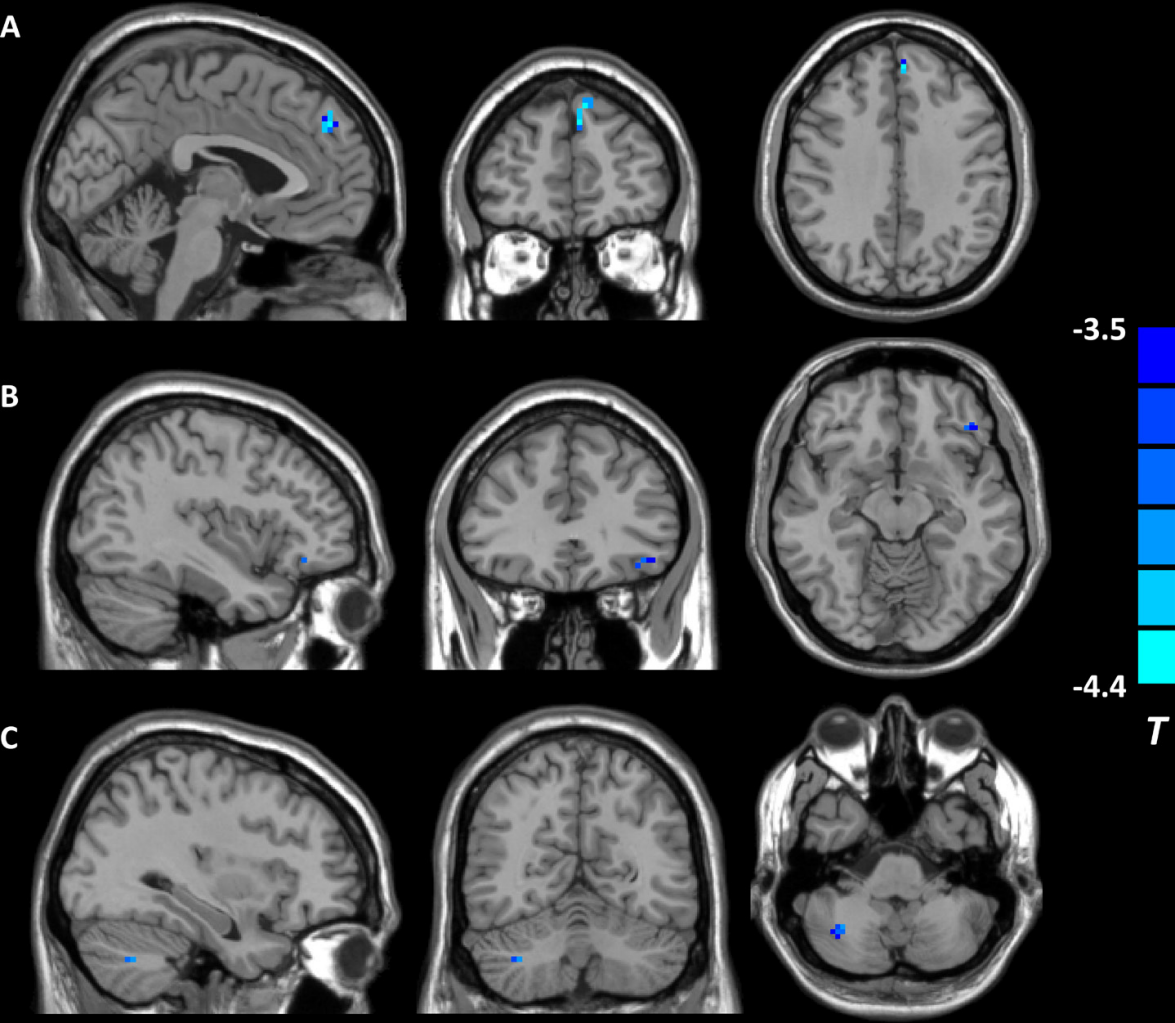
Brain regions showing decreased right precuneus-based functional connectivity in individuals reporting childhood trauma (*p* < 0.05, AlphaSim corrected). (**A**) the left ventromedial prefrontal cortex; (**B**) the left orbitofrontal cortex; (**C**) the right cerebellum.

The fALFF analysis revealed that individuals with childhood trauma showed altered brain activity in two DMN regions. Furthermore, fALFF values in the right precuneus, a core hub of DMN, exhibited significant between-group difference and correlated with CTQ scores in the whole sample. Therefore, the right precuneus (14 voxels) was selected as seed region for subsequent DMN connectivity analysis. Subjects with childhood trauma showed decreased FC in the left ventromedial prefrontal cortex (vmPFC), left orbitofrontal cortex (OFC), and right cerebellum (see Table 4 and Figure 2). Altered right precuneus-based FC was negatively correlated with CTQ scores, including CTQ total, emotional neglect, and physical neglect (*r* = −0.520 ∼ −0.700, *p* < 0.05, AlphaSim corrected) in the whole sample. In addition, a positive correlation between WCST completed categories and precuneus-based FC in the left vmPFC, as well as negative associations of preservative errors in WCST with precuneus-based FC in the left vmPFC and left OFC were found in the total sample (see Table 5).

**Table 4:** Brain regions showing reduced functional connectivity with the right precuneus in CTE individuals as compared with subjects without CTE

Brain region	Hemisphere	Cluster Size	*T* value	MNI coordinate		
				*x*	*y*	*z*
vmPFC	L	25	−4.29	−3	48	36
OFC	L	13	−3.84	−39	33	−12
Cerebellum	R	15	−4.06	33	−60	−42

(*p* < 0.05, AlphaSim corrected). CTE, Childhood Trauma Exposures; FC, Functional Connectivity; MNI, Montreal Neurological Institute; OFC, Orbitofrontal Cortex; vmPFC, Ventromedial Prefrontal Cortex; PE, Preservative Errors.

**Table 5:** Correlation analyses between executive function and the right precuneus-based functional connectivity in the whole sample

Brain region	Hemisphere	Cluster Size	*T* value	MNI coordinate			*r*
				*x*	*y*	*z*	
WSCT PE							
OFC	L	11	−4.02	−48	24	−9	−0.518
vmPFC	L	14	−4.16	−6	45	42	−0.556
WSCT Categories							
vmPFC	L	14	4.63	−9	45	42	0.579

(*p* < 0.05, AlphaSim corrected). FC, Functional Connectivity; OFC, Orbitofrontal Cortex; PE, Preservative Errors; vmPFC, Ventromedial Prefrontal Cortex; WSCT, Wisconsin Card Sorting Test.

## DISCUSSION

The present study investigated the executive performance and resting-state brain functional changes as measured by fALFF and seed-based FC analyses in young healthy adults with and without early trauma exposures. The current results revealed a significantly damaged executive function and aberrant brain regional activity and interregional FC within the DMN in subjects with childhood trauma, and furthermore, a significant association between executive deficits and diminished DMN connectivity in the whole sample. These outcomes together with previous findings in rodent and nonhuman primate studies [26] might help us understand the neuromechanisms underlying executive dysfunction resulting from chronic early life stress.

Cognitive disturbance has been increasingly recognized as an important aspect of childhood stress, characterized by poor performance on measures of executive function, processing speed, and working memory [27]. The present finding that individuals with childhood trauma show impaired executive function is consistent with the results of previous studies. For instance, Hanson et al. (2013) reported that early neglect experiences were associated with pervasive executive deficits in children raised in institutional settings [28]. And similarly, Nikulina et al. (2013) found that childhood maltreatment, especially childhood neglect, might be an important influence in the development of the executive function deficits of middle adulthood [29]. Moreover, childhood trauma has also been demonstrated to have detrimental effects on performance of neurocognitive tasks which are specially designed to evaluate important aspects of executive domains [30, 31]. In general, all these findings suggest that childhood trauma might adversely impact the development of cognitive systems that support executive functioning.

A second noteworthy finding of the current study was altered fALFF in two DMN regions, the right precuneus and STG, in subjects with childhood trauma. The precuneus, a major posterior node of the DMN, is proved to be involved in theory of mind, self-processing, social cognition, and episodic memory retrieval [32]. While the STG is implicated in receptive and nonverbal auditory and language processing [33], and is also thought to be involved in the processing of emotion [34]. Our data is generally comparable to a recent report in patients with schizophrenia showing an association between childhood trauma and activation of the precuneus when performing a Theory-of-Mind task [35]. The present finding is, at least partially, consistent with prior neuroimaging modalities, which have demonstrated altered spontaneous activity of the precuneus in combat related posttraumatic stress disorder (PTSD) [36] and typhoon sur*vivo*rs [37]. Beyond the precuneus, regional dysfunction in the STG which is assessed by using resting-state ReHo has already been reported in adults with early life stress [16]. Moreover, Hein et al. (2017) performed a quantitative meta-analysis to examine the effects of child maltreatment on brain function and found that the STG was hyperactive in maltreated individuals relative to controls when responding to threatening emotion [38]. Taken together, we speculate that altered fALFF in the precuneus and STG might contribute to specific disturbances in cognitive and emotional processing in individuals with childhood trauma, which may reflect risk for the development of trauma-related disorders [39].

Using a seed-based connectivity approach, the present study also demonstrated diminished resting-state DMN connectivity associated with childhood trauma in subjects even without psychiatric symptoms or medical illness. Our result of decreased precuneus-PFC (including vmPFC and OFC) connectivity among healthy adults reporting histories of childhood trauma is generally in line with past findings. Bluhm et al. (2009) first revealed an association between patients with chronic PTSD related to early life trauma and disrupted DMN connectivity showing decreased FC of posterior cingulate cortex (PCC) with mPFC [10]. Then, in two recent studies, altered connectivity between PCC and mPFC was observed in healthy adults with early life stress [9] and infants with higher interparental conflict since birth [40], respectively, both demonstrating the relationship of childhood trauma with DMN dysfunction. Furthermore, the current finding also provides new evidence to the hypothesis that early life adversity may disrupt the posterior-to-anterior connectivity which is established as part of the normal developmental process [41]. Our study builds on the existing literatures, and suggests that aberrant DMN function at rest may be an independent reflection of early trauma exposure.

Finally, and most importantly, this study confirmed a linear relationship of childhood trauma related executive deficits with disrupted DMN connectivity as revealed by decreased precuneus-vmPFC and precuneus-OFC connectivity, potentially elucidating the neural mechanism underlying executive manifestations of individuals with early trauma. The putative DMN is a task-negative network measured by resting-state fMRI, which includes mainly the medial temporal, frontal, and parietal cortical areas [42]. DMN is suggested to be closely related to higher cognitive functions [43] and this association has been extensively studied in patients with AD. Celebi et al. (2016) found that DMN activity was altered in AD and PCC functional connectivity with other parts of DMN was related to cognitive function scores, such as, memory, attention, and executive function [44]. More interestingly, a recent study in patients with amnestic mild cognitive impairment further demonstrated that accompanied by the ameliorative DMN connectivity, the cognitive performance improved significantly after two-year treatment [45], suggesting a possible relationship between these two aspects. Additionally, it was also shown that among trauma-exposed patients with MDD, higher scores on measures of verbal memory and executive functioning were related to increased connectivity within the DMN [46]. Owing to these findings and ours, it is believed that altered DMN connectivity is involved in disturbances of executive processing.

This study had several limitations. First, the sample size in each group was relatively small which restricted us to compare the different effects of five types of childhood trauma on brain activity. Second, the present FC analysis was limited to precuneus-based DMN connectivity only while ignoring other brain networks that were related to childhood trauma. Third, it was conceivable that childhood trauma had impact on several cognitive domains, however, only executive function was assessed in this study. Finally, this study was cross-sectional designed, which restricted causal analysis. Nevertheless, our novel findings may provide a new insight into exploring the neural underpinnings of childhood trauma. Previous studies have revealed that childhood trauma is associated with biological alterations, such as dysregulation of the hypothalamo-pituitary-adrenal (HPA) axis [47] and inflammation system [48], and interestingly, such disturbances may also contribute to brain abnormalities [49, 50]. In this context, future longitudinal studies containing the above mentioned aspects may be helpful to elucidate the detailed pathway underlying childhood trauma related manifestations. In addition, animal studies should further explore if childhood trauma is causally related to neural differences and subsequently cognitive deficits.

In summary, the present findings suggest that childhood trauma is associated with executive deficits and aberrant DMN functions even in healthy adults. In addition, this study demonstrates that executive dysfunction is related to disrupted DMN connectivity.

## MATERIALS AND METHODS

### Participants

The study group comprised 48 subjects (male/female, 18/30), ages 18-33 years, including 24 subjects with childhood trauma experiences (CTE group) and 24 age- and gender-matched subjects without childhood trauma exposures (non-CTE group). For assignment to the CTE group, individuals must have had experienced chronic moderate-severe trauma exposures (abuse or/and neglect) before the age of 16. All participants were recruited from a survey that we had carried out to investigate the occurrence of childhood trauma in local communities and universities. Subjects responded with no direct reference to childhood trauma as a key variable in the study. All subjects were thoroughly interviewed by two professional psychiatrists and were free from any current or lifetime history of psychiatric disorders according to Diagnostic and Statistical Manual of Mental Disorders, IV Edition (DSM- IV) criteria, as screened with the Structured Clinical Interview for DSM-IV interview (SCID). The general exclusions were as follows: (1) left handedness, (2) standard scores > 50 on SDS [51] or > 40 on SAS [52], (3) significant medical illness, (4) presence of major sensorimotor handicaps, (5) history of seizures, head trauma, or unconsciousness, (6) intake of any psychotropic medication or hormone, (7) alcohol or substance abuse, (8) women with pregnancy/lactating or in their menstrual period, (9) contraindications to MRI scan, including metallic implants, retractors or braces, and claustrophobia, and (10) inability to keep still during MRI scanning. The demographic data were collected using a self-designed questionnaire from all the participants. This study was approved by the ethic committee of the Second Xiangya Hospital of Central South University. A complete description of the study was provided to every subject, after that written informed consent was obtained from each participant.

#### Assessment of childhood trauma

The CTQ is a reliable and valid self-reporting questionnaire with 28 items [53]. It can yield five sub-scales which evaluate five aspects of CTE, emotional abuse, emotional neglect, sexual abuse, physical abuse, and physical neglect, respectively. Existence of CTE is determined by cutoff score of each CTQ sub-scale. Subjects who score higher than the threshold of a sub-scale are treated as existence of corresponding CTE. The cutoffs of each sub-scale for moderate-severe exposure are as follows: 1) emotional abuse ≥ 13, 2) emotional neglect ≥ 15, 3) sexual abuse ≥ 8, 4) physical abuse ≥ 10, and 5) physical neglect ≥ 10. The Chinese version of CTQ was introduced in our study. It was translated into Chinese by Zhao et al. (2005) [54], the CTQ has good internal consistency (Cronbach’s alpha) for the CTQ total score (0.77) and the five subscales range from 0.41 to 0.68 in a Chinese sample of 794 individuals. It has been widely used in many Chinese studies although many researchers would like to revise the CTQ and hope its subscales to be more suitable for the Chinese population [55].

#### Neuropsychological tests

A well-known neuropsychological test battery on executive function was administered to each subject which included WCST [56], SCWT [57], and TMT [58].

1) WCST: Subjects were asked to sort 48 cards on the basis of three possible categories (color, number, and shape). After six consecutive correct responses, subjects were asked to change the sorting principle to another category. The test ended when subjects completed all six categories correctly or used all 48 cards. We evaluated the primary efficacy outcome by using indices of WCST for the performance of executive function that included total trials (TT), total correct (CT), total errors (TE), preservative errors (PE), random errors (RE), and categories completed.

2) SCWT: In part A, the subjects were asked to read randomized color names printed in black type. Then, in part B, the subjects were required to name the color of dots. Finally, in part C, the subjects were instructed to name the ink color of a color word which was not the same as the ink color. All the subjects would have 45 seconds in each part. The performance for each condition was calculated by the finished amounts. The difference in part B relative to part C is called the ‘interference’ effect. SCWT was conducted to measure selective attention/processing speed (SCWT-A and SCWT-B), behavioral inhibition (SCWT-C), and executive function (SCWT interference).

3) TMT: In TMT-A, the subject was required to quickly draw lines to connect consecutively numbered circles. In TMT-B, the subject was asked to alternately combine numbers with letters in ascending order. The task completion is measured in seconds. Part A measured visuo-spatial attention and performance speed, whereas Part B required mental flexibility, ability to shift attention, and strategy.

#### MRI acquisition

The imaging data were obtained on a 3.0T Philips Achieva scanner at the Second Xiangya Hospital, Central South University. Subjects were asked to lie on the scanner and keep eyes closed. A standard birdcage head coil was used, and the restraining foam pads were placed on two sides of the head to minimize head motion while cotton plug was used with the purpose of diminishing the noise. A total of 180 volumes of echo planar images were obtained axially, the parameters were as follows: repetition time = 3,000 ms; echo time = 30 ms; slices = 36; thickness = 4 mm, no slice gap; field of view = 240 × 240 mm^2^; resolution = 64 × 64; flip angle = 90°; scan time = 9’09’’.

#### Data preprocessing

The MRIConvert program (http://lcni.uoregon.edu/∼jolinda/MRIConvert/) was used to convert neuroimaging data to Neuroimaging Informatics Technology Initiative data format. All the rs-fMRI data were processed using Data Processing Assistant for Resting-State fMRI Advanced Edition V2.2 (DPARSFA, http://www.restfmri.net/forum/DPARSF). The functional images were conducted for slice acquisition correction and head motion correction. The fMRI data which had less than 1.0 mm of head motion and 1.0° of angular rotation were included. Moreover, the mean framewise displacement (FD) was computed by averaging FDi from every time point for each subject [59]. There was no difference for the mean FD between two groups (*t* = 0.835, *p* = 0.408) (Table 1). Then the fMRI images were normalized to the standard Montreal Neurological Institute (MNI) template provided by Statistical Parametric Mapping (SPM) 8 and resample to the 3-mm isotropic voxels. The normalized images were smoothed using a 4-mm full width at half maximum (FWHM) Gaussian kernel. A temporal filter (0.01 Hz < f < 0.08 Hz) were used to reduce the low frequency drift and physiological high frequency respiratory and cardiac noise, and finally, the linear trend was removed.

#### fALFF and FC calculation

The measurement of fALFF was performed as described previously by Zou et al. (2008) [23]. The time series for each voxel was firstly transformed to the frequency domain using a Fast Fourier Transform (FFT), and then computing the sum of frequencies in the low frequency band (0.01–0.08 Hz). The ALFF measure at each voxel was the averaged square root of the power in the 0.01–0.08 Hz window, normalized by the mean within-brain ALFF value for that subject. This averaged square root was taken as the amplitude of LFF. The fALFF value was the ratio of the power spectrum of low-frequency (0.01–0.08 Hz) to that of the entire frequency range.

A seed-based interregional FC analysis was used in the present study. Areas where between-group fALFF differences correlated with childhood trauma were selected as seeds. The average time series data from seed regions were extracted and FC analysis was performed using the data resulting from preprocessing. The nuisance covariates, including cerebrospinal fluid signals, global mean signals, white matter signals, as well as six head motion parameters were regressed out; then a voxel-wise correlation analysis was conducted between the seed region and the rest of the brain (r score); and finally, Fisher’s r to z transformation was performed to improve the normality of the correlation coefficient.

#### Statistical analysis

Demographic and clinical data were analyzed by using Statistical Package for the Social Sciences version 16.0 (SPSS Inc., Chicago, IL, USA). Independent two-sample *t* tests and Chi-square tests (χ^*2*^) were respectively used to tests for the continuous variables and categorical variables between two groups. Values are given as mean ± standard deviation. The level of two-tailed statistical significance was set at *p* < 0.05 for all tests.

Between-group differences of fALFF and FC were analyzed using two-sample *t*-tests on a voxel-by-voxel basis with SPM8 software. The *t* map was set at a corrected significance level of *p* < 0.05 [combined height threshold *p* < 0.001 (*T* > 3.28) and cluster ≥ 6]. Threshold correction was performed by using AlphaSim program (parameters were as follows: individual voxel *p* = 0.001, 1000 simulations, FWHM = 4 mm, with mask) in the REST software, which applied Monte Carlo simulation to calculate the probability of false-positive detection by taking into consideration both the individual voxel probability thresholding and cluster size [60].

Furthermore, to evaluate any correlations of between-group fALFF or FC differences with CTQ and cognitive scores, whole brain multiple regression analyses integrated in SPM basic models were performed at *p* < 0.05 (AlphaSim corrected). Then, the mean fALFF and FC values of the survived clusters were extracted by using region of interest (ROI) analyses. Correlations were conducted using Pearson’s product moment.
